# Serotonin and Depression: A Disconnect between the Advertisements and the Scientific Literature

**DOI:** 10.1371/journal.pmed.0020392

**Published:** 2005-11-08

**Authors:** Jeffrey R Lacasse, Jonathan Leo

## Abstract

Many ads for SSRI antidepressants claim that the drugs boost brain serotonin levels. Lacasse and Leo argue there is little scientific evidence to support this claim.

In the United States, selective serotonin reuptake inhibitor (SSRI) antidepressants are advertised directly to consumers [1]. These highly successful direct-to-consumer advertising (DTCA) campaigns have largely revolved around the claim that SSRIs correct a chemical imbalance caused by a lack of serotonin (see Tables 1 and 2). For instance, sertraline (Zoloft) was the sixth best-selling medication in the US in 2004, with over $3 billion in sales [2] likely due, at least in part, to the widely disseminated advertising campaign starring Zoloft's miserably depressed ovoid creature. Research has demonstrated that class-wide SSRI advertising has expanded the size of the antidepressant market [3], and SSRIs are now among the best-selling drugs in medical practice [2].

**Table 1:**
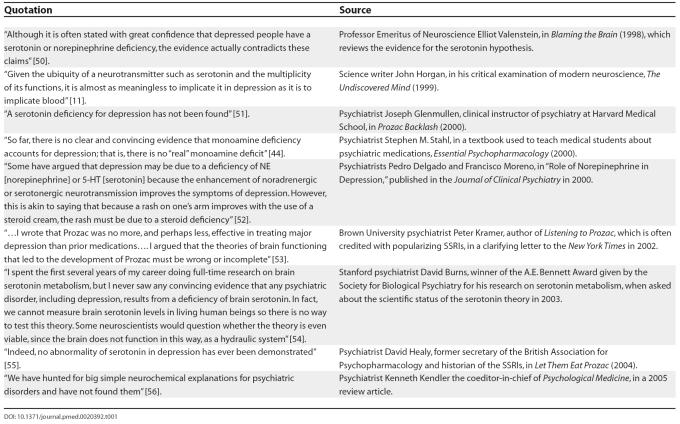
Selected Quotations Regarding Serotonin and Antidepressants

**Table 2:**
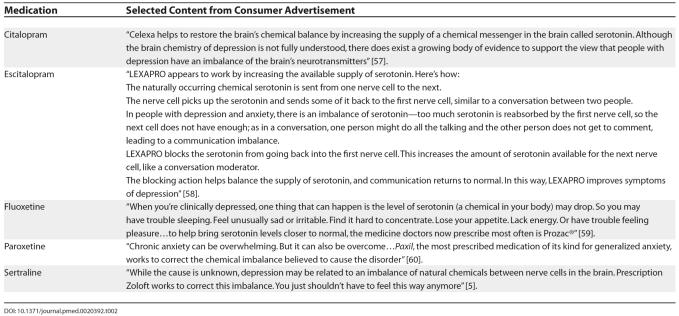
Selected Consumer Advertisements from SSRIs from Print, Television, and the World Wide Web

Given the multifactorial nature of depression and anxiety, and the ambiguities inherent in psychiatric diagnosis and treatment, some have questioned whether the mass provision of SSRIs is the result of an over-medicalized society. These sentiments were voiced by Lord Warner, United Kingdom Health Minister, at a recent hearing: “…I have some concerns that sometimes we do, as a society, wish to put labels on things which are just part and parcel of the human condition”[4]. He went on to say, “Particularly in the area of depression we did ask the National Institute for Clinical Excellence [an independent health organisation that provides national guidance on treatment and prevention] to look into this particular area and their guideline on depression did advise non-pharmacological treatment for mild depression” [4]. Sentiments such as Lord Warner's, about over-medicalization, are exactly what some pharmaceutical companies have sought to overcome with their advertising campaigns. For example, Pfizer's television advertisement for the antidepressant sertraline (Zoloft) stated that depression is a serious medical condition that may be due to a chemical imbalance, and that “Zoloft works to correct this imbalance” [5]. Other SSRI advertising campaigns have also claimed that depression is linked with an imbalance of the neurotransmitter serotonin, and that SSRIs can correct this imbalance (see Table 2). The pertinent question is: are the claims made in SSRI advertising congruent with the scientific evidence?

## The Serotonin Hypothesis

In 1965, Joseph Schildkraut put forth the hypothesis that depression was associated with low levels of norepinephrine [6], and later researchers theorized that serotonin was the neurotransmitter of interest [7]. In subsequent years, there were numerous attempts to identify reproducible neurochemical alterations in the nervous systems of patients diagnosed with depression. For instance, researchers compared levels of serotonin metabolites in the cerebrospinal fluid of clinically depressed suicidal patients to controls, but the primary literature is mixed and plagued with methodological difficulties such as very small sample sizes and uncontrolled confounding variables. In a recent review of these studies, the chairman of the German Medical Board and colleagues stated, “Reported associations of subgroups of suicidal behavior (e.g. violent suicide attempts) with low CSF–5HIAA [serotonin] concentrations are likely to represent somewhat premature translations of findings from studies that have flaws in methodology” [8]. Attempts were also made to induce depression by depleting serotonin levels, but these experiments reaped no consistent results [9]. Likewise, researchers found that huge increases in brain serotonin, arrived at by administering high-dose L-tryptophan, were ineffective at relieving depression [10]. [Fig pmed-0020392-g001]

**Figure pmed-0020392-g001:**
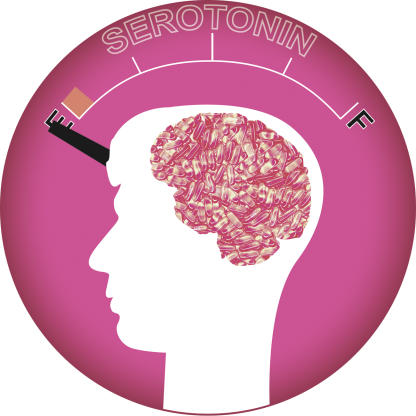
(Illustration: Margaret Shear, Public Library of Science)

Contemporary neuroscience research has failed to confirm any serotonergic lesion in any mental disorder, and has in fact provided significant counterevidence to the explanation of a simple neurotransmitter deficiency. Modern neuroscience has instead shown that the brain is vastly complex and poorly understood [11]. While neuroscience is a rapidly advancing field, to propose that researchers can objectively identify a “chemical imbalance” at the molecular level is not compatible with the extant science. In fact, there is no scientifically established ideal “chemical balance” of serotonin, let alone an identifiable pathological imbalance. To equate the impressive recent achievements of neuroscience with support for the serotonin hypothesis is a mistake.

With direct proof of serotonin deficiency in any mental disorder lacking, the claimed efficacy of SSRIs is often cited as indirect support for the serotonin hypothesis. Yet, this *ex juvantibus* line of reasoning (i.e., reasoning “backwards” to make assumptions about disease *causation* based on the response of the disease to a *treatment*) is logically problematic—the fact that aspirin cures headaches does not prove that headaches are due to low levels of aspirin in the brain. Serotonin researchers from the US National Institute of Mental Health Laboratory of Clinical Science clearly state, “[T]he demonstrated efficacy of selective serotonin reuptake inhibitors…cannot be used as primary evidence for serotonergic dysfunction in the pathophysiology of these disorders” [12].

Reasoning backwards, from SSRI efficacy to presumed serotonin deficiency, is thus highly contested. The validity of this reasoning becomes even more unlikely when one considers recent studies that even call into question the very efficacy of the SSRIs. Irving Kirsch and colleagues, using the Freedom of Information Act, gained access to all clinical trials of antidepressants submitted to the Food and Drug Administration (FDA) by the pharmaceutical companies for medication approval. When the published and unpublished trials were pooled, the placebo duplicated about 80% of the antidepressant response [13]; 57% of these pharmaceutical company–funded trials failed to show a statistically significant difference between antidepressant and inert placebo [14]. A recent Cochrane review suggests that these results are inflated as compared to trials that use an active placebo [15]. This modest efficacy and extremely high rate of placebo response are not seen in the treatment of well-studied imbalances such as insulin deficiency, and casts doubt on the serotonin hypothesis.

Also problematic for the serotonin hypothesis is the growing body of research comparing SSRIs to interventions that do not target serotonin specifically. For instance, a Cochrane systematic review found no major difference in efficacy between SSRIs and tricyclic antidepressants [16]. In addition, in randomized controlled trials, buproprion [17] and reboxetine [18] were just as effective as the SSRIs in the treatment of depression, yet neither affects serotonin to any significant degree. St. John's Wort [19] and placebo [20] have outperformed SSRIs in recent randomized controlled trials. Exercise was found to be as effective as the SSRI sertraline in a randomized controlled trial [21]. The research and development activities of pharmaceutical companies also illustrate a diminishing role for serotonergic intervention—Eli Lilly, the company that produced fluoxetine (Prozac), recently released duloxetine, an antidepressant designed to impact norepinephrine as well as serotonin. The evidence presented above thus seems incompatible with a specific serotonergic lesion in depression.

Although SSRIs are considered “antidepressants,” they are FDA-approved treatments for eight separate psychiatric diagnoses, ranging from social anxiety disorder to obsessive-compulsive disorder to premenstrual dysphoric disorder. Some consumer advertisements (such as the Zoloft and Paxil Web sites) promote the serotonin hypothesis, not just for depression, but also for some of these other diagnostic categories [22,23]. Thus, for the serotonin hypothesis to be correct as currently presented, serotonin regulation would need to be the cause (and remedy) of each of these disorders [24]. This is improbable, and no one has yet proposed a cogent theory explaining how a singular putative neurochemical abnormality could result in so many wildly differing behavioral manifestations.

In short, there exists no rigorous corroboration of the serotonin theory, and a significant body of contradictory evidence. Far from being a radical line of thought, doubts about the serotonin hypothesis are well acknowledged by many researchers, including frank statements from prominent psychiatrists, some of whom are even enthusiastic proponents of SSRI medications (see Table 1).

However, in addition to what these authors say about serotonin, it is also important to look at what is *not* said in the scientific literature. To our knowledge, there is not a single peer-reviewed article that can be accurately cited to directly support claims of serotonin deficiency in any mental disorder, while there are many articles that present counterevidence. Furthermore, the *Diagnostic and Statistical Manual of Mental Disorders (DSM)*, which is published by the American Psychiatric Association and contains the definitions of all psychiatric diagnoses, does not list serotonin as a cause of any mental disorder. The *American Psychiatric Press Textbook of Clinical Psychiatry* addresses serotonin deficiency as an unconfirmed hypothesis, stating, “Additional experience has not confirmed the monoamine depletion hypothesis” [25].

## Consumer Advertisements of Antidepressants

Contrary to what many people believe, the FDA does not require preapproval of advertisements. Instead, the FDA monitors the advertisements once they are in print or on the air [26]. Misleading content is frequently found in various DTCA campaigns [27]; hence, it is valuable to compare SSRI advertisements to the scientific evidence reviewed above. These SSRI ads are widely promulgated; hundreds of millions of dollars have been spent disseminating these advertisements, and one study found that over 70% of surveyed patients reported exposure to antidepressant DTCA [28].

## The Role of the FDA

In the US, the FDA monitors and regulates DTCA. The FDA requires that advertisements “cannot be false or misleading” and “must present information that is not inconsistent with the product label” [27]. Pharmaceutical companies that disseminate advertising incompatible with these requirements can receive warning letters and can be sanctioned. The Irish equivalent of the FDA, the Irish Medical Board, recently banned GlaxoSmithKline from claiming that paroxetine corrects a chemical imbalance even in their patient information leaflets [29]. Should the FDA take similar action against consumer advertisements of SSRIs?

As just one example, the prescribing information for paroxetine, which is typical of the SSRI-class drugs, states, “The efficacy of paroxetine in the treatment of major depressive disorder, social anxiety disorder, obsessive-compulsive disorder (OCD), panic disorder (PD), generalized anxiety disorder (GAD), and post-traumatic stress disorder (PTSD) is presumed to be linked to potentiation of serotonergic activity in the central nervous system resulting from inhibition of neuronal reuptake of serotonin. Studies at clinically relevant doses in humans have demonstrated that paroxetine blocks the uptake of serotonin into human platelets” [30].

In other words, the mechanism of action of paroxetine has not been definitively established, and remains unconfirmed and presumptive (the prescribing information states that the efficacy of the drug “is *presumed* to be linked to potentiation of serotonergic activity” ([30], our italics added). Although there is evidence that paroxetine inhibits the reuptake of serotonin, the significance of this phenomenon in the amelioration of psychiatric symptoms is unknown, and continually debated [12,31]. Most importantly, the prescribing information does not mention a serotonin deficiency in those administered paroxetine, nor does it claim that paroxetine corrects an imbalance of serotonin. In contrast, the consumer advertisements for paroxetine present claims that are not found in this FDA-approved product labeling.

In order to determine whether these advertisements actually comply with FDA regulations, it is useful to consult the Code of Federal Regulations under which DTCA is regulated. The regulations state that an advertisement may be cited as false or misleading if it “[c]ontains claims concerning the mechanism or site of drug action that are not generally regarded as established by scientific evidence by experts qualified by scientific training and experience **without disclosing that the claims are not established and the limitations of the supporting evidence…”** ([32], our emphasis added]).

Stating that depression *may* be due to a serotonin deficiency is seemingly allowed, but, as stated in the regulations, only if the limitations of the supporting evidence are provided. In our examination of SSRI advertisements, we did not locate a single advertisement that presented any such information. Instead, the serotonin hypothesis is typically presented as a collective scientific belief, as in the Zoloft advertisement, which states that regarding depression, “Scientists believe that it could be linked with an imbalance of a chemical in the brain called serotonin” [33]. Consumers viewing such advertisements remain uninformed regarding the limitations of the serotonin hypothesis (reviewed above).

According to federal regulations, advertisements are also proscribed from including content that “contains favorable information or opinions about a drug previously regarded as valid but which have been rendered invalid by contrary and more credible recent information” [32].

This means that a disconnect between the evolving peer-reviewed literature and advertisements is not permitted. Regarding SSRIs, there is a growing body of medical literature casting doubt on the serotonin hypothesis, and this body is not reflected in the consumer advertisements. In particular, many SSRI advertisements continue to claim that the mechanism of action of SSRIs is that of correcting a chemical imbalance, such as a paroxetine advertisement, which states, “With continued treatment, *Paxil* can help restore the balance of serotonin…” [22]. Yet, as previously mentioned, there is no such thing as a scientifically established correct “balance” of serotonin. The take-home message for consumers viewing SSRI advertisements is probably that SSRIs work by normalizing neurotransmitters that have gone awry. This was a hopeful notion 30 years ago, but is not an accurate reflection of present-day scientific evidence.

The FDA has sent ten warning letters to antidepressant manufacturers since 1997 [34–43], but has never cited a pharmaceutical company for the issues covered here. The reasons for their inaction are unclear but seem to result from a deliberate decision at some level of the FDA, rather than an oversight. Since 2002, the first author (JRL) has repeatedly contacted the FDA regarding these issues. The only substantive response was an E-mail received from a regulatory reviewer at the FDA: “Your concern regarding direct-to-consumer advertising raises an interesting issue regarding the validity of reductionistic statements. These statements are used in an attempt to describe the putative mechanisms of neurotransmitter action(s) to the fraction of the public that functions at no higher than a 6th grade reading level” (personal communication, 2002 April 11).

It is curious that these advertisements are rationalized as being appropriate for those with poor reading skills. If the issues surrounding antidepressants are too complex to explain accurately to the general public, one wonders why it is imperative that DTCA of antidepressants be permitted at all. However, contrary to what the FDA seems to be implying, truth and simplicity are not mutually exclusive. Consider the medical textbook, *Essential Psychopharmacology*, which states, “So far, there is no clear and convincing evidence that monoamine deficiency accounts for depression; that is, there is no ‘real’ monoamine deficit” [44]. Like the pharmaceutical company advertisements, this explanation is very easy to understand, yet it paints a very different picture about the serotonin hypothesis.

## Conclusion

The impact of the widespread promotion of the serotonin hypothesis should not be underestimated. Antidepressant advertisements are ubiquitous in American media, and there is emerging evidence that these advertisements have the potential to confound the doctor–patient relationship. A recent study by Kravitz et al. found that pseudopatients (actors who were trained to behave as patients) presenting with symptoms of adjustment disorder (a condition for which antidepressants are not usually prescribed) were frequently prescribed paroxetine (Paxil) by their physicians if they inquired specifically about Paxil [45]; such enquiries from actual patients could be prompted by DTCA [45].

What remains unmeasured, though, is how many patients seek help from their doctor because antidepressant advertisements have convinced them that they are suffering from a serotonin deficiency. These advertisements present a seductive concept, and the fact that patients are now presenting with a self-described “chemical imbalance” [46] shows that the DTCA is having its intended effect: the medical marketplace is being shaped in a way that is advantageous to the pharmaceutical companies. Recently, it has been alleged that the FDA is more responsive to the concerns of the pharmaceutical industry than to their mission of protecting US consumers, and that enforcement efforts are being relaxed [47]. Patients who are convinced they are suffering from a neurotransmitter defect are likely to request a prescription for antidepressants, and may be skeptical of physicians who suggest other interventions, such as cognitive-behavioral therapy [48], evidence-based or not. Like other vulnerable populations, anxious and depressed patients “are probably more susceptible to the controlling influence of advertisements” [49].

In 1998, at the dawn of consumer advertising of SSRIs, Professor Emeritus of Neuroscience Elliot Valenstein summarized the scientific data by concluding, “What physicians and the public are reading about mental illness is by no means a neutral reflection of all the information that is available” [50]. The current state of affairs has only confirmed the veracity of this conclusion. The incongruence between the scientific literature and the claims made in FDA-regulated SSRI advertisements is remarkable, and possibly unparalleled.

## Abbreviations

DTCA: direct-to-consumer advertising
FDA: Food and Drug Administration
SSRI: selective serotonin reuptake inhibitor
